# The Proposed New Medical Degree for London Students

**Published:** 1887-04

**Authors:** 


### *THE PROPOSED NEW MEDICAL DEGREE FOR LONDON STUDENTS.*

The new year finds the scheme for providing an attainable degree for London medical students much more nearly within the range of practical politics than it was twelve months ago. Since then, delegates from the College of Physicians and the College of Surgeons have held innumerable sittings, and presented a report, the most important part of which has now been accepted by both the colleges. The latest transaction in regard to it took place at a meeting of the College of

Physicians in December, when the report of the delegates was under discussion. It is quite possible that the report might have been rejected had it not been for the eloquent speech of Sir Henry Pitman, who smoothed away all objections with singular ability. The great argument of the objectors has always been that colleges cannot give degrees, that being the function of a university; and, therefore, they say, let a university be formed in the management of which the two colleges may have a share. But Sir Henry Pitman first told us that the University of Edinburgh existed as a college for a hundred years before becoming a university, and then gave, perhaps, even a better instance in the recent foundation in London of a Royal College of Music, which has the power to grant degrees in music; it is, therefore, clear that the university-theory objection can no longer be sustained. Another objection that was frequently raised was that the identity and existence of the College of Physicians would be merged in this new body; but those who urged this forgot that the combination of the two colleges had already taken place as far as is necessary, without in any way producing detriment to the individuality of either of them. The meeting ended with a practically unanimous decision to join with the College of Surgeons in presenting a petition to the Privy Council asking for power to confer a degree. What the exact terms are upon which this degree should be given, have yet to be settled; but the only point upon which there is likely to be any difference of opinion is as to whether it should be a *sine qua non* that the student shall have studied at one of the metropolitan schools of medicine. It appears to me that it should be, and for this simple reason—viz., that one of the chief reasons, if not the very foremost reason, for demanding a degree in London is the immense opportunity for clinical study which the metropolis affords, and to give this new degree to men who have not availed themselves of those opportunities would make it appear that the efficient training of the student was not an all-important point. It seems possible that the College of Physicians may differ with the College of Surgeons on this point, as it is rumored that the latter does not wish to insist upon any part of the study being of necessity carried out in London; but I hope it will be brought to see the error of its way, as I know that by many at the College of Physicians the matter will be regarded and treated as a vital one.—*N. Y. Medical Journal.*

